# Expanding on the use of The International Classification of Functioning, Disability and Health: Examples and resources

**DOI:** 10.4102/sajp.v78i1.1614

**Published:** 2022-08-17

**Authors:** Soraya Maart, Catherine Sykes

**Affiliations:** 1 Department of Health and Rehabilitation Sciences, Faculty of Health Sciences, University of Cape Town, Cape Town, South Africa; 2 School of Health Sciences, Faculty of Medicine and Health, University of Sydney, Sydney, Australia

**Keywords:** CF, framework, clinical practice, evidence, coding

## Abstract

Most physiotherapists today are familiar with the abbreviation ICF and know that it stands for the International Classification of Functioning, Disability and Health; the title of the World Health Organization’s international standard for describing health and health related states. Most universities in South Africa and globally have adopted the framework of the ICF in their curricula especially in promoting clinical reasoning, however, translating that knowledge for recording and monitoring has been slow in coming. Less well known, is how the ICF is and can be used and how it can inform physiotherapy practice, research, education and administration. Our article outlines the importance of the ICF to physiotherapy and recommends resources to facilitate expanded ICF use by physiotherapists. Examples are given of how to use coding and the importance of aggregating data and concluding with resources that can assist with the expanded use. Sufficient evidence and resources are available to support the expanded use of the ICF for data collection and clinical coding.

**Clinical implications:**

The aggregation of data can be used for the monitoring of universal health coverage especially in the context of National Health Insurance implementation.

## Introduction

Most physiotherapists today are familiar with the ICF framework and know that the acronym stands for the International Classification of Functioning, Disability and Health; the title of the World Health Organization’s international standard for describing health and health-related states (WHO 2001). Globally and in South Africa, physiotherapy programmes have adopted the framework of the ICF in their curricula especially in promoting clinical reasoning, however, translating that knowledge for recording and monitoring has been slow in coming. Less well known is how the ICF is and can be used and how it can inform physiotherapy practice, research, education and administration; although there is no doubt that the ICF has changed the way health and disability are perceived and monitored (Madden & Bundy 2019). Our article outlines the importance of the ICF to physiotherapy and recommends resources to facilitate expanded ICF use by physiotherapists.

Most diseases and health conditions manifest as an interruption or difficulty in functioning and resolution of this is recorded as the experience of an individual to function independently within their context. We need to be able to record that functioning, impact and monitor changes over time. That is where the ICF comes in (WHO 2001).

## What is classification and why is it important?

Rachel Hurst a representative of Disabled People International during the development of the ICF clarified the controversy around the need for classification, not of people, but of their situation:

[*I*]n a perfect world we would prefer to have no classification at all… however, for the purposes of statistics, assessment for services and programmes and above all for non-discrimination legislation, we do need to have a definition of who we are and of our situation…. (Rachel Hurst, Disabled Peoples International 1998)

Classification is part of the human condition (Bowker & Star 2000). Classification is a process for ordering information; something everyone does in their day to day lives, socks in one part of the closet and shirts in another.

Classification is for a purpose, for example, weights for boxing competitions and age for access to schools. Patients seen by physiotherapists are generally organised according to body system affected (musculoskeletal, neurology, respiratory), age (paediatrics, geriatrics) or sex (women’s health, men’s health). We might classify in a further level of detail for disease type (arthritis, stroke, asthma), part of the body (shoulder injury, low back pain, acquired brain injury) or location of practice (acute care, rehabilitation, primary care, private practice). Classification is often contentious, as was seen in the case of Caster Semenya’s eligibility for running in races, where the classification by male or female genetics was challenged by a classification by testosterone levels.

## Why collect data?

Data can help you improve and inform a range of activities including service delivery, planning and evaluation audit or research, advocacy and understanding the need for a particular group, area or practice. Data can help you answer questions about the characteristics of the community you serve, whether changes to government or organisational policies have resulted in changes in the service you provide or for the people in your care or whether your practice or the people you serve are similar or different from other groups. Maart et al. (2007) demonstrated that in two areas of South Africa, although the level of impairment across areas was similar, the experience of environmental barriers was different. More people with disabilities experienced barriers with access to Public Buildings e150 in the Western Cape compared with the Eastern Cape. Similarly, Attitudes of Society e460 was a bigger barrier in the Eastern Cape (Maart et al. 2007). The recording of this type of information is vital in the development and monitoring of comprehensive services within a country. In the development of the ICF, its value was perceived in the ability to record and monitor functioning across the lifespan and the effectiveness of interventions at individual or societal levels (Ustün et al. 2003).

## Why use the International Classification of Functioning, Disability and Health?

Physiotherapy is about changing the functioning of our patients and or clients. The ICF provides the means to record human functioning regardless of the health conditions diagnosed, it is a tool with universal application. It organises information across all facets of functioning and the environmental factors that are inextricably linked to the level of functioning. The ICF functioning is seen as the product of the interaction between the environment, a person’s personal factors and any health conditions a continuous variable, labelled disability when a certain cut-off point along the continuum is reached. That cut point is set according to purpose such as eligibility for and equity of access to services or determining discrimination. This is the role of governments.

The framework of the ICF can be used to organise your assessment, to recognise what is important to the person and note what is not collected. The arrows in the framework are bidirectional. Analysing the relationships using clinical reasoning can help us to devise and prioritise goals; not only impairment goals (change in muscle strength for example) but also participation goals (how the change in muscle strength improves participation). After all it is usually a change in participation or functioning that brings a patient to see a physiotherapist. You can collect consistent and reliable data to generate information about your practice, your patients and your outcomes.

In a clinical scenario the progress of functioning can be recorded through the use of qualifiers to indicate the level of impairment using a 5-point scale (1 = no impairment, 2 = mild, 3 = moderate, 4 = severe and 5 = complete). Moderate difficulty with grasping objects in the task of opening a jar can be coded as d4401.3.

At a practice level, data generated using the ICF in clinical assessment can be aggregated to give information about a group. For example, how many people in your practice have a problem with doing housework (d640) that can be aggregated from all the 4 digit categories, which may be collected differently across practices. Data can also be aggregated for combined intervention based on the context of the functional limitation, for example, how many persons are struggling to walk on uneven surfaces d4502 post stroke. This could inform the rehabilitation structure at your practice.

Importantly, aggregated and de-identified data can be used for reporting on a local, regional, national or international level. Myezwa et al. (2011) were able to collate data collections across countries and languages to generate new information about HIV. A key message in data collection is ‘collect once and use often’. The ICF can be used as the framework for national data collections, the common language for a more consistent understanding of functioning and disability. The hierarchical structure of the codes to aggregate detailed data, for example, data collected in the clinical encounter should be processed to more consolidated form in order to provide sound health information and decision making.

It is possible to build up a profile of functioning over time. An injury can change the profile of functioning and the recovery monitored through the reacquisition of functions. The profile of functioning changes throughout life and it may be possible to estimate the need for support as certain functions change. For example, as grip strength declines (b7300 Power of isolated muscles and muscle groups) the need for an assistive device for opening jars may be indicated (e 1151 Assistive products and technology for personal use in daily living) but also the need for support with other activities (d440 Fine hand use) and participation (relevant codes from Chapters 7 [Education], 8 [Work and employment] and 9 [Community, social and civic life]) may be predicted (Bohannon 2015).

## Using the International Classification of Functioning, Disability and Health for outcomes monitoring

In a recent WHO exercise the ICF was found to be useful in tracking universal health coverage (UHC) indicators. The need for physiotherapists to now expand their use of the ICF beyond the framework into coding has become imperative for the tracking and global comparability of UHC and Sustainable Development Goals (SDGs).

### Tracking of universal health coverage indicators

**TABLE 1:** Tracking of universal health coverage indicators using the International Classification of Functioning, Disability and Health.

100 core health indicators + health-related SDGs (based on 2018 update)	WHO-FIC		
	ICD-10	ICD-11	ICF
Stillbirth rate	X	X	-
New cases of vaccine-preventable diseases	X	X	-
Incidence of low birth weight among newborns	X	X	X
Total alcohol per capita (age 15+ years) consumption (SDG 3.5.2)	X	X	X
HIV test results for TB patients	X	X	-
Coverage of essential health services (SDG 3.8.1)	-	-	X
Perioperative mortality rate	X	X	-
Birth registration (SDG 16.9.1)	X	X	X

Source: Whitelaw, L., Almborg, A., Martenuzzi, A. & Van Gool, C., 2021, ‘Update on the mapping of the global reference list of 100 core health indicators to the three WHO-FIC reference classifications’, *WHOFIC Virtual Annual Meeting 18–22 October 2021*, viewed 06 December 2021, from https://cdn.who.int/media/docs/default-source/classification/who-fic-network/meetings/who-fic-network-virtual-annual-meeting-2021/whofic_booklet_poster_2021.pdf?sfvrsn=bba58ef9_5

UHC, universal health coverage; ICF, International Classification of Functioning, Disability and Health; SDG, Sustainable Development Goals; HIV, human immunodeficiency virus; TB, tuberculosis; ICD, International Classification of Disease; WHO-FIC, The World Health Organisation Family of International Classifications Network.

### Example of a specific service coverage indicator

**TABLE 2:** Service coverage indicator using the International Classification of Functioning, Disability and Health. Coverage of diarrhoea treatment.

ICD-10	ICD-11	ICF
A09.- (Other gastroenteritis and colitis of infectious and unspecified origin)	ME05.1 (Diarrhoea)	b525 (Defecation functions)
K52.- (Other noninfective gastroenteritis and colitis)	DD91.2 (Functional diarrhoea)	b530 (Weight maintenance function)
		b545 (Water, mineral and electrolyte balance functions)
		e1101 Medications (changed title accepted 2020)

*Source:* Whitelaw, L., Almborg, A., Martenuzzi, A. & Van Gool, C., 2021, ‘Update on the mapping of the global reference list of 100 core health indicators to the three WHO-FIC reference classifications’, *WHOFIC Virtual Annual Meeting 18–22 October 2021*, viewed 06 December 2021, from https://cdn.who.int/media/docs/default-source/classification/who-fic-network/meetings/who-fic-network-virtual-annual-meeting-2021/whofic_booklet_poster_2021.pdf?sfvrsn=bba58ef9_5

ICF, International Classification of Functioning, Disability and Health; ICD, International Classification of Disease.

The reporting and coding of low birth weights in paediatric practice can provide the prevalence of these codes for monitoring. The inclusion of Chapter V in the ICD 11 should encourage other health professionals to capture functioning as part of routine assessment and management. This should make the use of ICF codes and framework more accessible to medical doctors.

## International Classification of Functioning, Disability and Health-based tools and resources to facilitate expanded use

### World Health Organization Disability Assessment Schedule

Designed for use in both clinical and population collections the World Health Organization Disability Assessment Schedule (WHODAS) 2.0 is a generic assessment instrument. It captures level of functioning in six domains of life: cognition, mobility, self-care, getting along, life activities and participation. There are 12 item and 36 item versions with the short version explaining 81% of the variance in the longer form. Administration may be by self-report, interview or by proxy (WHO 2010). World Health Organization Disability Assessment Schedule 2.0 has been validated across cultures and is widely used globally. Translations have been made into isiXhosa, Afrikaans and Zulu amongst others (Federici et al. 2016). The WHODAS has effectively been used in countries for disability registration and compensation (Salinas-Rodríguez et al. 2020). The ability to explore more than the impairment, as is currently the use in the South African census, provides a more nuanced appreciation of disability.

### International Classification of Functioning, Disability and Health checklist

The ICF checklist is a practical tool to elicit and record information on functioning at an individual level. It is a clinician completed form which includes the major categories of ICF and some personal factors (WHO 2003). It should be noticed that the checklist has not been updated and so should be checked against the most recent version of the ICF in the online browser. The ICF checklist has been criticised for being too long and including items that may not be relevant for a discreet population, such as those with HIV (Myezwa et al. 2009). It has also been successfully used to describe environmental barriers experienced by people with disability in rural and urban areas in South Africa (Maart et al. 2007).

### World Health Organization Model Disability Survey

The Model Disability Survey (MDS) is a standardised instrument for data collection on disability for understanding of how people live their lives and the identification of key barriers to full participation, for prevalence estimates for tracking national trends and making international comparisons, for analysis of the interaction between health conditions and environmental factors and for monitoring the situation of people with disability. The data can be used in policymaking. There are a brief version and a long version that can be used in either a household or individual capacity.

### International Classification of Functioning, Disability and Health core sets

To facilitate the use of the ICF core sets have been developed (eds. Bickenbach et al. 2021). The core sets are selected categories specific to a certain health condition or health service situation. The selections were made following a scientific process and validated (Selb et al. 2015). An online interactive ICF-based Documentation Tool (https://www.icf-core-sets.org) enables the user to develop forms based on ‘core sets’ of ICF by providing lists of relevant categories selected from the classification.

### How to use the International Classification of Functioning, Disability and Health: A practical manual for using the International Classification of Functioning, Disability and Health

Through a series of questions, the ICF Practical Manual provides an outline of why the ICF should be used, how to get started and examples of uses to describe functioning, in clinical practice and the education of health professionals, for community support services and income support, for population-based census or survey data, in education systems, for policy and programme purposes and for advocacy and empowerment purposes. A key message from this document is that data collected once, might have multiple purposes.

### International Classification of Functioning, Disability and Health e-learning tool

The WHO and its collaborating centres have developed an online self-learning tool introducing users to the ICF, its philosophy, structure and use (https://www.icf-elearning.com/).

### Call to action

Our article presents a call to physiotherapists to use the ICF coding to collect data. Specific examples have been provided as to how this could be performed and its benefits in tracking UHC indicators and the possible development of clinical guidelines.

With further development of the electronic platform of the WHOFIC, the joint use of diagnosis (ICD), intervention (ICHI) and functioning outcome (ICF) will hopefully facilitate holistic reporting of patient care. The data can be analysed to inform practice, policy and advocacy (Madden & Bundy 2018).

Contact the South African Collaborating Centre http://www.whofic.org.za for more information.
